# Prospective, longitudinal, multi-modal functional imaging for radical chemo-IMRT treatment of locally advanced head and neck cancer: the INSIGHT study

**DOI:** 10.1186/s13014-015-0415-7

**Published:** 2015-05-15

**Authors:** Liam Welsh, Rafal Panek, Dualta McQuaid, Alex Dunlop, Maria Schmidt, Angela Riddell, Dow-Mu Koh, Simon Doran, Iain Murray, Yong Du, Sue Chua, Vibeke Hansen, Kee H. Wong, Jamie Dean, Sarah Gulliford, Shreerang Bhide, Martin O. Leach, Christopher Nutting, Kevin Harrington, Kate Newbold

**Affiliations:** The Royal Marsden NHS Foundation Trust, Downs Road, Sutton, Surrey SM2 5PT UK; The Institute of Cancer Research, 15 Cotswold Road, Sutton, Surrey SM2 5NG UK; Clinical Research Fellow, Head and Neck Unit, Royal Marsden Hospital, Sutton, Surrey SM2 5PT UK

**Keywords:** Hypoxia, Imaging, MRI, PET, Radiotherapy, Head and neck cancer

## Abstract

**Background:**

Radical chemo-radiotherapy (CRT) is an effective organ-sparing treatment option for patients with locally advanced head and neck cancer (LAHNC). Despite advances in treatment for LAHNC, a significant minority of these patients continue to fail to achieve complete response with standard CRT. By constructing a multi-modality functional imaging (FI) predictive biomarker for CRT outcome for patients with LAHNC we hope to be able to reliably identify those patients at high risk of failing standard CRT. Such a biomarker would in future enable CRT to be tailored to the specific biological characteristics of each patients’ tumour, potentially leading to improved treatment outcomes.

**Methods/design:**

The INSIGHT study is a single-centre, prospective, longitudinal multi-modality imaging study using functional MRI and FDG-PET/CT for patients with LAHNC squamous cell carcinomas receiving radical CRT. Two cohorts of patients are being recruited: one treated with, and another treated without, induction chemotherapy. All patients receive radical intensity modulated radiotherapy with concurrent chemotherapy. Patients undergo functional imaging before, during and 3 months after completion of radiotherapy, as well as at the time of relapse, should that occur within the first two years after treatment. Serum samples are collected from patients at the same time points as the FI scans for analysis of a panel of serum markers of tumour hypoxia.

**Discussion:**

The primary aim of the INSIGHT study is to acquire a prospective multi-parametric longitudinal data set comprising functional MRI, FDG PET/CT, and serum biomarker data from patients with LAHNC undergoing primary radical CRT. This data set will be used to construct a predictive imaging biomarker for outcome after CRT for LAHNC. This predictive imaging biomarker will be used in future studies of functional imaging based treatment stratification for patients with LAHNC. Additional objectives are: defining the reproducibility of FI parameters; determining robust methods for defining FI based biological target volumes for IMRT planning; creation of a searchable database of functional imaging data for data mining. The INSIGHT study will help to establish the role of FI in the clinical management of LAHNC.

**Trial registration:**

NCRI H&N CSG ID 13860

## Background

Head and neck cancer (HNC) is the fifth most common cancer worldwide [1]. Most HNC patients receive multimodality therapy combining surgery, radiotherapy and chemotherapy in an attempt to eradicate disease whilst preserving organ function. At least 50 % of HNC patients present with stage III/IV disease, defined as locally advanced head and neck cancer (LAHNC) [1]. In these patients, there has been a shift away from surgery towards organ-preserving treatment protocols involving concomitant cisplatin-based chemo-radiotherapy [2]. When compared to surgical treatment, chemo-radiotherapy delivers equivalent, or better, loco-regional control and disease-free survival, but with significantly improved functional outcomes [2, 3].

Despite advances in treatment techniques, LAHNC continues to have disappointing 5-year disease-free and overall survival rates of 30–40 % [1]. Strategies to improve outcomes by escalating conventionally-delivered radiotherapy and/or cytotoxic chemotherapy appear attractive but pose significant risks of severe acute and late normal tissue damage and threaten chronic structural, cosmetic and functional deficits that will have a significant negative impact on quality of life [4–6]. Importantly, recent technological developments in physical targeting of radiation delivery, including intensity-modulated and image-guided radiotherapy, now offer a means of selectively escalating dose to tumour tissue without exceeding normal tissue tolerances [7, 8].

Biological studies have recently characterised LAHNC as a disease spectrum, divisible into different prognostic groups, based on demographic (tobacco exposure), clinical/radiological (T and N stage) and molecular pathological (HPV status) variables [9]. We can now look beyond the standard model in which all patients receive treatment according to a “one size fits all” philosophy. We can, instead, look forward to rational treatment individualisation according to biologically defined prognostic risk stratification. To achieve this, we require the ability to stratify patients with LAHNC by means of predictive biomarkers, which would ideally provide reliable information about the likely impact of a specific therapeutic intervention [10]. Predictive biomarkers that rely on tissue biopsies are limited by sampling bias due to intra-tumour heterogeneity [11, 12]. In contrast, functional imaging techniques offer the possibility of characterising much more completely, the spatio-temporal heterogeneity of tumours, in terms of parameters that are known to have biological significance, such as those characterising tumour perfusion, oxygenation, and metabolism [13, 14]. By using functional imaging techniques, it may be possible to identify specific areas of intrinsic radio-resistance within tumours, and use these areas as targets for radiation dose-escalation [8, 15–17]. Alternatively, functional imaging parameters measured at baseline and during early treatment may be able to function as reliable biomarkers for treatment response prediction and allow for subsequent treatment selection [14, 18–21].

A common theme in published studies of functional imaging techniques in HNC is a lack of consensus as to how best to analyse the resulting data to extract clinically relevant signals pertaining to the tumour and its response (or otherwise) to treatment [14, 22–24]. A yet more complex question is how best to combine data and parameters from different functional imaging modalities to maximise the combined predictive power of these techniques [14, 24, 25]. The goal of functional imaging predictive biomarker studies is to identify patients for specific treatment strategies in order to improve treatment outcomes. In LAHNC, such strategies could include: radiotherapy dose escalation to specific tumour volumes (e.g. hypoxia) identified on functional imaging, treatment with specific targeted drugs e.g. anti-EGFR therapy [26–28] or DNA repair inhibitors [29–31], or treatment de-escalation in the case of patients with favourable functional imaging profiles [32, 33].

Treatment response assessment using functional imaging requires that an observed change in a parameter during or after treatment be greater than the intrinsic variability of the parameter in the absence of treatment. In the absence of treatment, HNC tumour size usually changes relatively slowly, and is reproducibly measurable [34]. By contrast, the physiologically dependent parameters measured by functional imaging techniques, such as perfusion, oxygenation, and glucose metabolism are intrinsically dynamic and are known to fluctuate, over varying timescales, in the absence of therapy [14]. An additional source of significant functional imaging parameter variability in HNC is the size and anatomical location of the primary tumour and the extent of regional lymph node metastases. These sources of variation have not typically been taken into account in published studies of functional imaging in HNC.

A further uncertainty is the optimal timing of functional imaging scans during the course of radical chemo-radiotherapy. A prospective study of 20 patients undergoing radical chemo-radiotherapy within the Head and Neck (H&N) Unit at The Royal Marsden Hospital (RMH) used weekly CT scanning during radiotherapy looked at tumour volume changes during treatment [34]. This study showed that the most significant changes in tumour volume during chemo-radiotherapy had already occurred by the end of the second week of radiotherapy [34]. A subsequent prospective pilot study of functional imaging in 10 patients undergoing radical chemo-radiotherapy within the H&N Unit at RMH showed that there are substantial changes in tumour tissue functional MRI and 18 F-fluorodeoxyglucose (FDG) PET/CT parameters after two cycles of induction chemotherapy, before radiotherapy has begun [35]. Out of 9 evaluable patients in the pilot functional imaging study, 7 achieved a complete metabolic response on FDG-PET/CT following two cycles of induction chemotherapy prior to starting radiotherapy. There were also significant reductions in the dynamic contrast-enhanced MRI (DCE-MRI) parameters (IAUGC60, and Ktrans), and a significant increase in the diffusion weighted imaging (DWI) apparent diffusion coefficient (ADC) parameter over the same period [35]. These results from our pilot study suggest that functional imaging data needs to be gathered early in the course of treatment for LAHNC, if important differences in tumour response to treatment are not to be missed. Additional findings supporting early data acquisition during treatment come from a prospective longitudinal study of serial 18 F-fluoromisonidazole (FMISO) PET/CT in 25 patients with LAHNC undergoing radical chemo-RT without induction chemotherapy [21]. This study found that FMISO-PET parameters derived from scans after 1 and 2 weeks of chemo-RT week had the strongest association with local-progression-free-survival.

There are numerous technical difficulties in characterising tumour hypoxia using FMISO-PET [36] and it would be preferable to find alternative, simpler, and less costly hypoxia imaging methods. Intrinsic susceptibility-weighted MRI exploits the paramagnetic properties of deoxyhaemoglobin in erythrocytes to create contrast and may be able to provide rapid imaging of tumour hypoxia with high spatial resolution. Deoxyhaemoglobin creates magnetic susceptibility perturbations around blood vessels and the transverse MR relaxation rate R_2_* (R_2_* = 1/T_2_*) of water in blood and the surrounding tissues increases in proportion to tissue deoxyhaemoglobin concentration [37, 38]. Deoxyhaemoglobin, therefore, is an intrinsic (endogenous), blood oxygenation level–dependent (BOLD) contrast agent. Because oxygenation of haemoglobin is proportional to arterial blood polarographic oxygen levels and, therefore, in equilibrium with tissue polarographic oxygen levels, tumour R_2_* is a sensitive index of tissue oxygenation and a surrogate marker of hypoxia [37]. BOLD-MRI has often been used together with an oxygen challenge [38], but this adds complexity to the scanning process, and may not be tolerable as part of a multi-modality functional MRI protocol. However, there are now data which suggest that useful information may still be obtained from BOLD-MRI images even in the absence of an oxygen challenge [39, 40].

The feasibility of using biological tumour volumes (BTVs) defined from functional MRI as targets for intensity-modulated radiotherapy (IMRT) dose escalation has been investigated in our pilot study of longitudinal functional imaging during radical chemo-IMRT for LAHNC [35]. Patients in this study received two cycles of induction chemotherapy (IC) prior to definitive radical chemo-radiation. Multi-parametric functional imaging including DCE and DWI showed marked biological responses making it difficult, or even impossible, to delineate small volume residual disease during radiotherapy planning and delivery following IC. Based on these data, such BTV boost volumes would have to be defined on the original baseline images acquired before IC. However, the physical and biological relevance of such BTVs, at the start of CRT, following 2 cycles of IC, is far from clear. The findings from our pilot study contrast with other studies in which FI was acquired during CRT without prior IC [18, 41–48]. In those studies, untreated tumour was visible on FI modalities at the outset of CRT and it was possible to implement adaptive radiotherapy strategies in treatment planning and phase 1 studies [44, 46, 49].

Following on from our pilot study of longitudinal functional imaging for radical chemo-IMRT for LAHNC [35], we have developed a successor study, termed INSIGHT (funct**I**onal imagi**N**g for defining biological radiotherapy target volume**S**, assessing disease response, and pred**I**cting loco-re**G**ional control in patients undergoing c**H**emo-radio**T**herapy for head and neck cancer). The INSIGHT study is gathering longitudinal multi-parametric functional imaging from two expanded cohorts of patients with LAHNC. The principal aim of the INSIGHT study is to develop a predictive functional imaging biomarker for response to chemo-IMRT treatment for LAHNC. Such a longitudinal functional imaging biomarker could be used as the basis of treatment selection for patients with LAHNC in future clinical trials aiming to personalise treatment by directing patients towards specific treatment protocols and interventions determined by the behaviour of their tumour during treatment.

## Methods/design

### Study organisation and funding

The INSIGHT study was designed through collaboration between the Head and Neck Unit and Dept. of Radiology of the Royal Marsden Hospital, and the Division of Radiotherapy and Imaging of the Institute of Cancer Research. The study sponsor is The Royal Marsden Hospital NHS Foundation Trust. All study investigations including FDG-PET/CT, functional MRI, and chemo-IMRT are undertaken within the Royal Marsden Hospital, London and Sutton. The study is funded in part by grants from Cancer Research UK (C46/A10588 and C7224/A1340), and additional funding is received via the NCRI H&N CSG. The study has received institutional review board approvals from the RMH Committee for Clinical Research (CCR study number 3926) and the National Research Ethics Committee (REC number 13/LO/0067). The study complies with the declaration of Helsinki, the British Good Clinical Practice (GCP) regulations, and the UK Data Protection Act. The study has been adopted into the NCRI Head and Neck Cancer research portfolio (NCRI UKCRN 13860) and is listed on the publicly accessible NCRI research study database.

### Study design and patient population

**Fig. 1:**
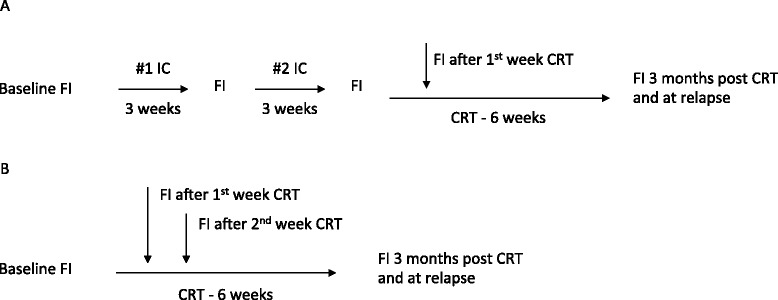
Timelines for INSIGHT study functional imaging (FI) scans before, during, and after radical chemo-radiotherapy. (**a**) Timing of FI for patients receiving two cycles of induction chemotherapy (IC) prior to primary radical chemo-radiotherapy (CRT). For this group, FI consists of the following scans: DCE-MRI, DW-MRI, BOLD-MRI, FDG-PET/CT at each time point, except during CRT when FDG-PET/CT is omitted. (**b**) Timing of FI for patients receiving primary radical chemo-radiotherapy (CRT) alone. For this group, FI consists of the following scans: DCE-MRI, DW-MRI, BOLD-MRI, FDG-PET/CT at each time point. For both groups repeated pre-treatment baseline FI scans will be performed in a proportion of patients. Blood tests for FBC and serum markers of hypoxia will be taken on the same day as each of the FI scans

INSIGHT is a prospective longitudinal observational cohort study at a single cancer centre, across two sites, of patients with locally advanced squamous cell carcinoma of the head and neck, receiving definitive primary treatment with radical chemo-IMRT. Patients may be treated with or without induction chemotherapy. All patients undergo serial 18 F-FDG PET/CT and multi-parametric functional MRI (DCE, DWI, and BOLD) before, during, and 3 months after completion of treatment (Fig. 1). Serum samples are also collected from patients at the time of each MRI scan. The serum will be used to measure the concentrations of a panel of serum markers of tumour hypoxia that have been shown to be relevant to treatment outcomes for LAHNC [50]. Circulating tumour DNA levels could also potentially be measured from the study serum samples, which may offer complementary data to assist in differentiating the responses of patient’s tumours to treatment [51, 52].

The study workflows for patients treated with and without induction chemotherapy are shown in Fig. 1. In addition to the scans at the time points shown in Fig. 1, patients with loco-regionally recurrent disease occurring within the first two years after treatment will undergo repeat 18 F-FDG PET/CT and multi-parametric functional MRI. The majority of loco-regional recurrences following treatment for LHANC occur in the first 2 years following treatment [53, 54], and recurrent disease occurring later than two years after primary treatment may represent a second primary cancer. For this reason follow-up imaging within the INSIGHT study is limited to recurrences occurring within two years of the end of primary chemo-radiotherapy.

### Inclusion criteria for the INSIGHT study are

1. Patients with documented histologically proven squamous cell carcinoma of the head and neck planned for primary radical chemo-radiotherapy, with or without induction chemotherapy followed by radiotherapy with concomitant platinum based chemotherapy.
2. Age > 18 years.
3. World Health Organisation (WHO) performance status 0 – 2.
4. Written informed consent.

### Exclusion criteria for the study are

1. WHO performance status >2.
2. Patients with any previous malignancy apart from non-melanoma skin cancer.
3. Patients with contraindications to MRI scanning.
4. Patients with contraindications to IV contrast agents.
5. Patients with renal failure [estimated or measured EDTA glomerular filtration rate (GFR) below 60 ml/min/1.73 m2].
6. Pregnancy or lactation.
7. Patient refusal.

### Study objectives

The principal aim of the INSIGHT study is to construct a risk scoring system based on functional imaging parameters to predict a patient’s response to radical chemo-radiotherapy and their probability of obtaining loco-regional disease control. This will be achieved by comparing changes in functional MRI and FDG-PET/CT parameters per patient in responders vs. non-responders as defined by clinical evidence of residual disease 3 months after completion of treatment. Secondary objectives are to:

1. Define reproducibility of the DCE, DWI, and BOLD MRI measurements for this study population.
2. To look for evidence of a stable functional imaging derived radiotherapy target volume (biological target volume) that could be subject to radiotherapy dose escalation.
3. To validate identified biological target volumes by means of comparison of functional imaging findings at baseline and during treatment with those at the time of relapse.
4. To explore correlation of serum levels of hypoxia biomarkers, and FI parameters before, during, and after radical chemo-radiotherapy.
5. To generate a database of functional and anatomical imaging for patients with head and neck cancers for evaluation of new radiotherapy strategies and planning techniques. The database will be explored using data mining techniques to look for novel patterns in functional imaging parameters across patients that may be of biological and clinical relevance [24].

### Chemotherapy

All patients are treated according to RMH Head and Neck Unit institutional protocols [7, 55, 56]. Patients receiving induction chemotherapy, are treated with either 2 cycles of TPF (docetaxel 75 mg/m2 day 1, cisplatin 75 mg/m2 day 1, 5-FU 750 mg/m2 days 1–4) or 2 cycles of CF (cisplatin 75 mg/m2 day1, 5-FU 1000 mg/m2 days 1–4). Concomitant chemo-IMRT is given with cisplatin 100 mg/m2 on days 1 and 29 of IMRT. For patients with a contraindication to cisplatin (EDTA GFR < 50 ml/min; hearing loss; tinnitus) carboplatin (AUC = 5) is substituted [57], but TPF is only given to patients able to receive cisplatin.

### IMRT

Patients undergo radiotherapy treatment planning in accordance with the protocols of the RMH Head and Neck Unit [53, 54]. After thermoplastic mask immobilisation a contrast-enhanced CT scan of the head and neck is obtained. Standard inverse planning techniques are used to plan treatment using either fixed field IMRT or VMAT according to the preference of the treatment planner and treating clinician. Treatment plans are developed using either the Eclipse (Varian Medical Systems, Palo Alto, USA) or Pinnacle (Philips Radiation Oncology Systems, Fitchburg, USA) treatment planning systems. FDG PET/CT and functional MRI data are not used for target delineation or dose prescription in this study. A dose of 65Gy in 30 fractions over 6 weeks is prescribed to the primary tumour PTV and involved lymph node levels, and 54Gy in 30 fractions over six weeks is prescribed to prophylactically treated regions. Tolerance doses of the brain stem, spinal cord, and optic apparatus are not exceeded. Parotid gland doses are minimised where feasible. Treatment is delivered on an outpatient basis, unless patients require hospital admission for supportive care.

### 18 F-FDG PET/CT

PET/CT studies are acquired using either a Gemini PET/CT (Philips, The Netherlands) or a Biograph mCT S128 (Siemens, Erlangen, Germany). Patients are asked to fast for at least 4 h before the study. 18 F-FDG (400 MBq) is injected intravenously if the blood sugar level is < 10 mmol/L. Patients rest for 60 min prior to PET/CT acquisition. Patients are positioned on a flat top couch and immobilised using a thermoplastic mask to match the position used for radiotherapy delivery. Emission data are acquired from the base of skull to carina. Unenhanced CT is performed from the base of skull to carina for purposes of attenuation correction and image fusion for anatomical localization. Quantitative image analysis of PET/CT data will be performed using Hermes [Hermes Medical Solutions, Stockholm, Sweden], and in-house software.

### Functional MRI

**Fig. 2:**
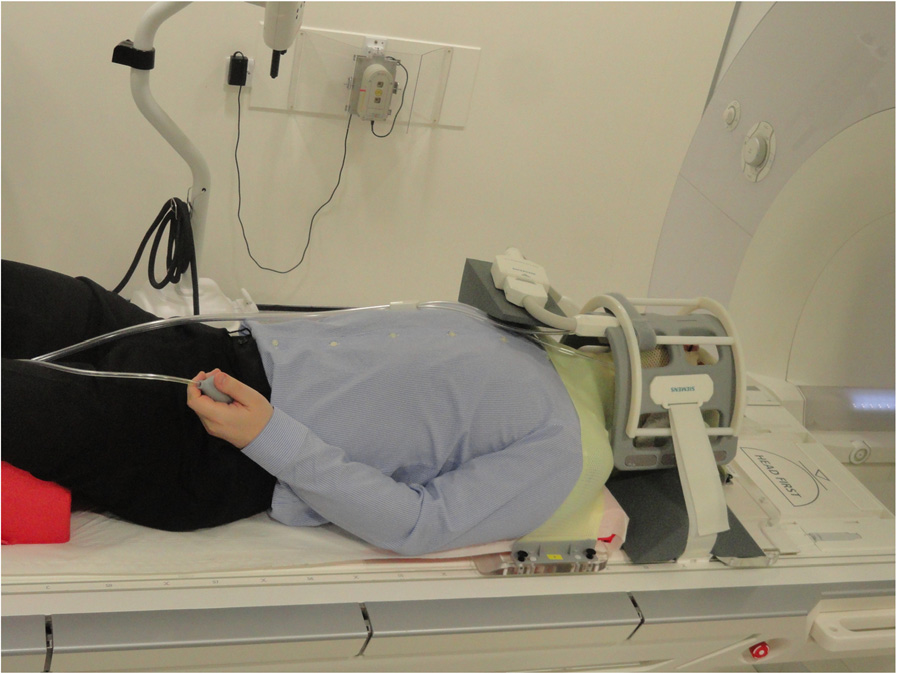
INSIGHT study MRI patient set up (photograph shows one of the study authors during study design and testing). Study patients are positioned on a flat top MRI couch using the same baseplate, neck rest, shoulder rest, and thermoplastic mask as used for radiotherapy treatment planning and delivery. A single multi-channel phased array flexible coil placed over the thermoplastic mask is used to provide anterior coverage of the anatomy of interest, whilst spine-array coils built-in to the couch provide posterior coverage. This set up allows for rapid patient positioning and MRI data acquisition in the radiotherapy treatment position which facilitates subsequent image co-registration with longitudinal MRI scans as well as with CT and PET/CT data

All functional MRI examinations are performed using clinical 1.5 T MRI scanners (Aera, Siemens Medical Solutions, Erlangen, Germany). As for PET/CT, patients are positioned to match the position used for radiotherapy delivery. A flat top MRI couch is used with an appropriate head rest and thermoplastic shell immobilisation. A single multi-channel phased array flexible coil is used to provide anterior coverage of the anatomy of interest (Fig. 2), whilst spine-array coils built-in to the couch provide posterior anatomical coverage. Non-contrast anatomical imaging is obtained first in order to assess the extent of the disease and aid functional MRI planning. Subsequently, and prior to contrast injection (in order to avoid changes in tissue relaxation), BOLD and DWI sequences are acquired over the volume of interest. The DCE-MRI sequence is obtained with bolus contrast injection (0.2 mL/kg body mass, Dotarem, Guerbet, France).

Functional MRI data will be processed using in-house software including the MRI Workbench (MRIW) [58], as well as additional in-house software developed using Matlab [The MathWorks, Inc., Natick, Massachusetts, United States]. Functional parameter maps (eg. K^trans^, ADC, R_2_*) will be exported in the DICOM format for use in radiotherapy treatment planning systems (Pinnacle, Philips Radiation Oncology Systems, Fitchburg, USA, and RayStation, RaySearch, Stockholm, Sweden).

### Analysis of blood samples

A panel of chemokines, cytokines and growth factors that have been shown to be associated with treatment outcomes for patients with LAHNC [Byers et al. [50], will be quantified for the isolated serum sample using a multiplex bead assay. Following Byers et al. [50], a panel of serum proteins will be measured at each study time point including: Eotaxin, Osteopontin, VEGF, IL-1β, IL-4, IL-8, IL-10, IL-12, IL-18, IFN-α, Gro-α, SDF-1α, FGF-1, TNF-α, TGF-β, PDGF, GCSF, HGF, MIF-1 and Leptin. Complete blood count analysis will be performed and haematocrit value used for DCE-MRI processing.

### Clinical follow-up

Following completion of treatment, patients will be followed up in accordance with standard practice within the RMH Head and Neck Unit. Patients undergo clinical examination including flexible nasendoscopy and contrast enhanced CT imaging of the head and neck 3 months following completion of treatment to determine therapeutic efficacy. Patients with evidence of residual or recurrent disease at this point will be deemed to have failed treatment, irrespective of whether or not they may be suitable for surgical salvage. Patients are subsequently seen at RMH for study specific follow-up on a 6-monthly basis for the first two years after treatment, in addition to standard clinical follow-up. Patients with evidence of relapse within the first two years undergo repeat FDG PET/CT and functional MRI, as for the other study time points.

### Evaluation of local response

Local response to chemo-IMRT is judged clinically, on examination, including flexible nasendoscopy. Radiological response is assessed on contrast enhanced CT according to RECIST criteria. Any patient with evidence of residual disease at 3 months post treatment will be deemed to have failed treatment and will considered for surgical salvage.

### Data handling, storage, and archiving

**Fig. 3:**
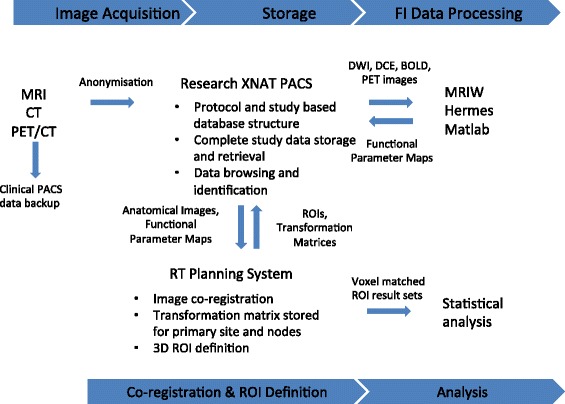
INSIGHT study data processing pathways. Anatomical and functional image data are acquired using RMH clinical systems and backed up on to the RMH clinical picture archiving and storage system (PACS). Anonymised image data are transferred electronically from the clinical PACS to a dedicated research PACS system, based on XNAT [56, 57]. Data are extracted from XNAT for subsequent processing, such as the generation of functional imaging parameter maps, and the results are stored within the same XNAT database. The XNAT system can communicate with the radiotherapy treatment planning systems (RTPS) that are used for multi-modality image co-registration and region-of-interest (ROI) volume definition. Voxel-wise functional image data from ROIs are extracted from image data using an RTPS and stored in XNAT from which they are then retrieved for subsequent statistical analysis

All imaging and radiotherapy data will be anonymised, coded and securely stored in a custom research PACS database built from the Extensible Neuroimaging Archive Toolkit (XNAT) open source imaging informatics platform [59, 60]. The XNAT system will be used to identify and retrieve image sets required for multimodality image co-registration, outlining of BTV ROIs (RT planning station) and functional imaging processing (MRIW, Matlab). The study data processing flow is illustrated in Fig. 3. All clinical data will be stored in accordance with British GCP regulations. The data will be kept under the custody of the PI with access only to authorised staff. Study documentation and medical records will be held for 5 years after study conclusion.

### Statistical analysis

#### Primary outcome

##### Response prediction

Multiple logistic regression analyses will be performed to test the independence of established clinical factors for the prediction of clinical complete remission at 3 months following completion of chemo-radiotherapy. Continuous variables (functional imaging parameters such as K^trans^, ADC, R_2_*, SUVmax) determined from DCE-MRI, DWI, BOLD-MRI, and FDG-PET/CT will be included in the initial multivariate model. Multivariable Cox regression analysis will be used to explore the relationship between functional imaging parameters and progression free survival (PFS) and overall survival (OS).

In order to determine which parameter or combination of parameters best predicts clinical response after chemo-radiotherapy, patients will be split (2:1) into a training set on which to build a predictive model, and a smaller test set on which to validate it. Receiver operating characteristic (ROC) curves for the prediction of residual disease after radical chemo-radiotherapy will be generated to determine cut-off values that yield optimal sensitivity and specificity. The differences in sensitivity, specificity, positive predictive value, negative predictive value, and accuracy will be compared using the McNemar test.

#### Other outcomes

##### Reproducibility of FI parameters

Reproducibility will be assessed using the Bland-Altman method [61–63]. Two pre-treatment baseline scans per patient will be compared. Absolute differences in a given functional imaging parameter between baseline scans are plotted against the mean of the two parameter measurements and reported as % change or absolute change depending on whether or not variability is independent of size of the measurement. If % change is more appropriate, the coefficient of variation (CV) will be calculated (half the absolute difference divided by the mean of the parameter) and mean and 95 % confidence limits for CV presented. If absolute change is more appropriate, limits of agreement (LoA) with confidence intervals (CI) will be reported showing the magnitude below which 95 % of differences can be expected to lie.

##### Response assessment

A repeated-measures multivariate analysis of variance (MANOVA) test will be used to provide a comparison of pre-, intra-, and post-treatment levels of functional imaging parameters at each time point. This will allow us to determine if there are significant differences in the changes in the functional imaging parameters during treatment in patients achieving complete response at 3 months and those with residual disease at that point.

##### Biological target volume

Cluster analysis methods will be applied to the functional imaging data to identify distinct sub-volumes within primary tumours and involved lymph nodes (BTVs). These methods will be applied to each of the distinct imaging modalities separately. We will look at consensus BTVs across each of the functional imaging modalities. These BTVs will be imported in to the radiotherapy treatment planning system to assess the coverage of the BTVs by the treatment dose distribution. We will explore the feasibility of radiotherapy dose escalation to identified BTVs by means of radiotherapy planning studies. To help validate BTVs identified on baseline and intra-treatment functional imaging scans, these BTVs will be correlated with the location and volume of loco-regional failure in patients who relapse during follow-up.

##### Serum markers

There is a lack of published literature on change in levels of cytokines and growth factors during the course of radical chemo-radiotherapy in patients with LAHNC, and therefore this aspect of the study will be exploratory. Distributions of serum marker variables according to baseline factors, and correlations between markers and functional imaging parameters will be assessed with exact non-parametric tests (Wilcoxon, Fisher, and Spearman). The levels of the serum markers of hypoxia, functional imaging parameters will be compared before, during, and after treatment using a multivariate repeated measures ANOVA test (MANOVA), after testing the data for normality.

##### Functional imaging database

All data gathered during this study will be stored within a single XNAT database. Known clinical characteristics with prognostic significance (e.g. HPV status), data derived from the measurement of serum markers of hypoxia, and data on outcomes following treatment will be stored in the XNAT database and associated with the stored scans for each patient. This will allow the database to be queried for ROIs with specific functional imaging characteristics to be associated with specific clinical features and treatment outcomes.

There will therefore be five data types per patient within the XNAT database:

1. potential prognostic variables (demographic and clinical variables that may influence outcome e.g. TNM stage, HPV status, smoking status, age, sex)
2. anatomical imaging data (CT and MRI) and the planned radical radiotherapy dose distribution
3. functional imaging data and associated parameters derived from these scans (e.g. K^trans^, v_e_, k_ep_, IAUGC60 and T_1_ from DCE-MRI; ADC values from DWI; R_2_* values from BOLD MRI; SUV values from FDG-PET/CT)
4. clinical outcomes (response to treatment at 3 months post-RT, 2-year PFS and 2-year OS)
5. serum concentration of tumour hypoxia markers

By storing all anatomical and functional imaging data together with data on known clinical characteristics, serum makers of hypoxia, and treatment outcomes, we will create a unique database that can be interrogated to look for novel associations between parameters that are associated with adverse treatment outcomes. This database will enable us to generate new hypotheses regarding functional imaging parameters and outcomes in HNC.

### Number of patients

**Fig. 4:**
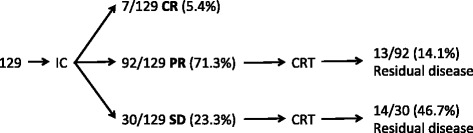
Response to induction chemotherapy and primary radical-chemoradiotherapy for 129 patients with locally advanced HNC treated in the H&N Unit at RMH from January 2001 to September 2006. Data taken from (Bhide et al. [55]) [53]. (IC: induction chemotherapy; CRT: chemo-radiotherapy; CR: complete response; PR: partial response; SD: stable disease)

Outcomes for patients with locally advanced HNC receiving standard treatment with induction chemotherapy and radical chemo-radiotherapy within the H&N Unit at RMH have been published [55]. These data show that after 2 cycles of induction chemotherapy, 5.4 % achieve a complete response (CR), 71.3 % a partial response (PR), and 23.3 % stable disease (SD) (according to RECIST1.0 criteria) (Fig. 4) [53]. Of those patients with PR and SD, 14.1 % and 46.7 % respectively will have clinical evidence of residual disease following completion of radical chemo-radiotherapy. Patients with residual disease after completion of treatment underwent neck dissection, and this revealed the presence of residual viable disease in 66.7 % [55]. According to these data, for every 100 LAHNC patients treated using the standard RMH H&N Unit regimen there will be 21 patients with clinical evidence of residual disease following completion of standard radical chemo-radiotherapy (10 having had PR with induction chemotherapy, and 11 having had SD). These are the patients we wish to identify earlier in the course of treatment by means of functional imaging in order to deliver alternative/escalated treatment to improve their treatment outcome.

Using functional imaging parameters derived from our pilot study [35] we have calculated estimates for the minimum numbers of patients required in order to be able to detect statistically significant differences in the pre- and post-chemotherapy values of K^trans^, ADC, and SUVmax between patients responding and not-responding to treatment. For a two-sided test at the 5 % significance level, for treatment induced changes in imaging parameters of 40 % (functional imaging responder) and 15 % (functional imaging non-responder) respectively, and with standard deviations for the imaging parameters of 30 % and 40 % respectively, a total of 66 patients will be required for a power of 80 % to predict response versus non-response on individual functional imaging parameters. This study is recruiting two such cohorts: one cohort of patients receiving induction chemotherapy, and another cohort of patients treated without induction chemotherapy.

Because this study obtains imaging data earlier during the course of treatment than did our pilot study, and also because of improvements in the imaging technology used for this study, there is a degree of uncertainty regarding the magnitude of the changes in functional imaging parameters that we will observe. These considerations mean that there is some uncertainty in the required sample size that is difficult to quantify. An interim data analysis (described below) will assist in quantifying the magnitude of changes in functional imaging parameters to be expected at each study time point. It is acknowledged that the sample size may need to be revised according to the results of this interim analysis as well as according to the rate of complete response to primary chemo-radiotherapy among recruited patients.

### Interim data analysis

An interim analysis will be undertaken after the first 10 patients have completed their scheduled study scans (up to and including the 3 month post-treatment scans). This will enable the relevance of the chosen scanning time points to be assessed and amendments made, if it appears that insufficient information is available from scanning at any given time point. A threshold for the minimum tumour volume of 0.63 cm^3^ has been derived from our pilot study for the functional MRI scans [35]. This minimum volume allows for a signal to be obtained from at least 20–40 voxels, which is a reasonable lower limit on the basis of our pilot study data. If we find that the average tumour volume for the first 10 patients at any time point in the study is less than 0.63 cm^3^ then the scans at that time point may be discontinued.

## Discussion

By gathering functional imaging data from a sizable cohort of patients with LAHNC undergoing radical chemo-radiotherapy we will build a database of tumours and their biological characteristics, as determined from functional imaging parameters. It may be possible to construct a scoring system, based on a combination of standard tumour characteristics and functional imaging parameters, to make an early prediction of treatment response and the probability of achieving loco-regional control after radical chemo-radiotherapy. If we can establish such a reliable functional imaging predictive biomarker for HNC patients we could use this to select patients for tailored treatments as part of a future randomised biomarker study in which patients were allocated to alternative treatments [64]. Such alternative treatments could include: intensification or de-escalation by modulating radiotherapy dose and/or by the addition or subtraction of concurrently delivered drugs including targeted agents [8, 14].

Prospective longitudinal functional imaging studies are essential to defining the place of these imaging techniques in clinical practice [21, 35, 65]. Despite being available as research tools for many years, functional imaging has yet to find a role within the standard clinical management of LAHNC. Studies such as INSIGHT that obtain data at multiple time points are needed to identify the optimal time points for distinguishing between patients that will achieve success versus those that will fail [21, 35]. Only then can rationally designed interventional functional imaging biomarker studies be established [14]. The INSIGHT study described here is an important step towards undertaking such biomarker driven interventional studies for patients with LAHNC. Prospective longitudinal imaging studies do require significant numbers of scans, as well as needing to recruit significant numbers of patients, in order to achieve a representative sample of the relevant patient population. Ideally, such studies would be conducted at multiple centres to facilitate rapid accrual of data, but there remain significant obstacles to performing multi-centre functional imaging studies, particularly in relation to DCE-MRI [66, 67]. These obstacles cannot be ignored if functional imaging is to form a routine part of patient management to permit treatment personalisation, as the results of functional imaging studies will need to be generalisable across treatment centres. We hope to expand the INSIGHT study into a multi-centre endeavour in due course, to help to achieve the much sought after goal of defining the utility of functional imaging in the management of LAHNC. Interested investigators are invited to contact the corresponding author.
